# The role and research progress of the balance and interaction between regulatory T cells and other immune cells in obesity with insulin resistance

**DOI:** 10.1080/21623945.2021.1876375

**Published:** 2021-01-20

**Authors:** Leiling Liu, Jiahui Hu, Yating Wang, Hao Lei, Danyan Xu

**Affiliations:** Department of Cardiovascular Medicine, The Second Xiangya Hospital, Central South University, Changsha, Hunan, China.

**Keywords:** Obesity, insulin resistance (IR), adipose tissue, regulatory T (Treg) cells

## Abstract

Metabolic homoeostasis in adipose tissue plays a major role in obesity-related insulin resistance (IR). Regulatory T (Treg) cells have been recorded to regulate metabolic homoeostasis in adipose tissue. However, their specific mechanism is not yet known. This review aims to present the role of Treg cells and other immune cells in obesity-associated IR, focusing on the balance of numbers and functions of Treg cells and other immune cells as well as the crucial role of their interactions in maintaining adipose tissue homoeostasis. Th1 cells, Th17 cells, CD8^+^ T cells, and pro-inflammatory macrophages mediate the occurrence of obesity and IR by antagonizing Treg cells, while anti-inflammatory dendritic cells, eosinophils and type 2 innate lymphoid cells (ILC2s) regulate the metabolic homoeostasis of adipose tissue by promoting the proliferation and differentiation of Treg cells. γ δ T cells and invariant natural killer T (iNKT) cells have complex effects on Treg cells, and their roles in obesity-associated IR are controversial. The balance of Treg cells and other immune cells can help maintain the metabolic homoeostasis of adipose tissue. Further research needs to explore more specific molecular mechanisms, thus providing more precise directions for the treatment of obesity with IR.

## Introduction

Although obesity with type 2 diabetes is the most common metabolic disease, its causes remain largely unclear [1]. Chronic, systemic low-grade inflammation is characteristic of obesity, type 2 diabetes, and other insulin resistance (IR) states [2,3]. Metabolic homoeostasis of adipose tissue helps maintain normal blood glucose levels, glucose tolerance, and insulin sensitivity, while chronic inflammation in adipose tissue leads to adipocyte hypertrophy and obesity-related IR [4]. In recent years, many immune cells residing in adipose tissues, such as regulatory T (Treg) cells, macrophages, dendritic cells, and CD4^+^ T cells, have been reported to be involved in regulating the homoeostasis and function of adipocytes [5].

**Figure 1.**
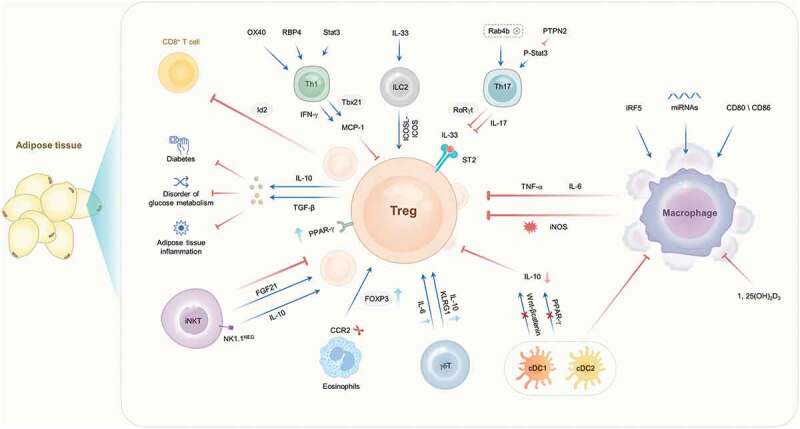
The molecular mechanisms by which the interactions between treg cells and various immune cells are regulated in obesity with IR. IR, insulin resistance; treg, regulatory T; ILC2s, type 2 innate lymphoid cells; iNKT, Invariant natural killer T; DCs, Dendritic cells; FOXP3, transcription factor forkhead box protein P3; IL, interleukin; TGF-β, transforming growth factor-β; RBP4, retinol-binding protein 4; IFN-γ, interferon-γ; MCP-1, monocyte chemoattractant protein-1; Stat3, signal transducer and activator of transcription 3; PTPN2, protein tyrosine phosphatase N 2; PPAR-γ, peroxisome proliferator-activated receptor-γ

Treg cells, an immunosuppressive subset of CD4^+^ T cells characterized by expression of the transcription factor forkhead box protein P3 (FOXP3), are immune regulatory cells [6] that can inhibit excessive inflammatory responses and maintain immune homoeostasis [7,8]. Developmental or functional defects in Treg cells can lead to uncontrolled immune responses and tissue destruction, which in turn leads to inflammatory diseases such as graft-versus-host disease, transplant rejection, and autoimmune diseases [9]. More importantly, functional defects of Treg cells are also closely related to the occurrence and development of obesity with IR [10], but the mechanism needs to be expounded. This review briefly analyzes and summarizes the roles of the balance and interaction of Treg cells with other T cell subsets, adaptive immune cells, and invariant natural killer T (iNKT) cells in obesity with IR (Table 1), to explore the cellular mechanism by which Treg cells regulate the metabolic homoeostasis of adipose tissue and provide new ideas for the treatment of obesity and obesity-related metabolic diseases (Figure 1).

**Table 1.** The balance and interaction between regulatory T cells and other immune cells in obesity with insulin resistance

Other immune cells	Methods	Number of other immune cells	Number of Treg cells	IR	Reference
Th1 cells	OX40-KO	↓	↑	↓	[25]
	T cell-sepecific Stat3-KO	↓	↑	↓	[26]
Th17 cells	High levels of insulin stimulation in vitro	↑	↓	↑	[35]
	T cell-specific Rab4b-KO	↑	↓	↑	[36]
	PTPN2 overexpression in EWAT	↓	↑	↓	[37]
CD8^+^ T cells	Treg cell-specific loss of Id2	↑	↓	↑	[53]
	Treg depletion in obese mice	↑	↓	↑	[12]
	Treg transfer in obese mice	↓	↑	↓	[12]
Macrophages	Treg transfer in B7-KO mice	↓	↑	↓	[66]
	IFN-γ and LPS stimulation in vitro	↑	↓	↑	[46]
Dendritic cells	specific knockdown of β-catenin in cDCs	↓	↓	↑	[72]
	specific KO of Pparγ in cDCs	↓	↓	↑	[72]
	activation of β-catenin in cDCs	↑	↑	↓	[73]
iNKT cells	αGalCer treatment in vitro	↑	↑	↓	[80]
	CD1d^−/-^ and Ja18^−/-^ (iNKT cell-deficient) mice	↓	↓	↑	[80]
	αGalCer treatment in vitro	↑	↓	↑	[82]
Eosinophils	CCR2^−/-^ mice	↑	↑	↓	[85]
γδ T cells	TCRδ^−/-^ mice	↓	↓	↓	[88]
ILC2s	deletion of OX40L on ILC2s (Il7ra^Cre/+^Tnfsf4^fl/fl^mice)	↓	↓	↑	[92]
	ILC2-deficient mice	↓	↓	↑	[93]

KO, knockout; OX40, T cell costimulatory molecule; stata3, signal transducer and activator of transcription 3; Rab4b, Ras-associated GTP-binding protein 4b; EAWT, epididymal white adipose tissue; Id2, transcriptional regulator; B7, CD80 and CD86; LPS, lipopolysaccharide; CCR2: the C-C chemokine receptor type 2; ILC2s, type 2 innate lymphoid cells.

Moreover, a small part of the research only discussed the changes in IR and immune cell phenotypes in obese patients, or only explored the impact of immune cells on IR in adipose tissue, but did not mention whether the above results occurred in the obesity models. Thus, it could not confirm changes in the IR and immune cells must be caused by obesity, but can only emphasize the correlations with obesity (Table 2). Although these studies can still provide ideas and directions for the treatment of obesity with IR, we should carefully interpret the results of the above studies.

**Table 2.** The relationship between changes in IR and inflammation phenotypes and changes in obesity

Reference	Model (s)	Whether it is associated with metabolic disorders	Whether changes in IR and inflammation phenotypes are primarily due to the changes in obesity
11	leptin deficient mice (Lep^ob/ob^ commonly referred to as ob/ob) A^y^/a mice mice fed HFD	IR	Yes
12	obese patients db/db leptin receptor deficient mice (BKS.Cg-Dock7m^+/+^Lepr^db^/J)	IR	Yes
15	Lep^ob/ob^ mice	IR	Yes
16	mice fed HFD	IR	Yes
17	mice fed HFD	IR	Yes
18	diet-induced obese C57BL/6NTac mice	IR	Yes
23	mice fed HFD	IR	Yes
24	homozygote type 2 diabetic mice (Lepr^db^) (Background Strain: C57BLKS/J)	IR	Yes
25	mice fed HFD	IR	Yes
26	mice fed HFD	IR	Yes
31	Lep^ob/ob^ mice fed HFD	IR	Yes
34	Lep^ob/ob^ mice fed HFD	IR	Yes
36	mice fed HFD	IR	Yes
37	mice fed HFD	IR	Yes
44	mice fed HFD	IR	Yes
45	mice fed HFD	IR	Yes
46	mice fed HFD	IR	Yes
47	mice fed HFD	IR	Yes
51	fragmented sleep-induced obese mice	IR	Yes
53	mice fed HFD	IR	Yes
58	obese/diabetic models ob/ob, db/db, agouti, and tubby in the C57BL/6 J strain fed HFD	IR	Yes
60	mice fed HFD	IR	Yes
61	mice fed HFD	IR	Yes
62	mice fed HFD	IR	Yes
65	monosodium L-glutamate (MSG) Wistar rats (newborn)	IR	Yes
66	mice fed HFD	IR	Yes
70	mice fed HFD	IR	Yes
72	mice fed HFD	IR	Yes
73	mice fed HFD	IR	Yes
76	mice fed HFD	IR	Yes
77	mice fed HFD	IR	Yes
78	mice fed HFD	IR	Yes
79	mice fed HFD	IR	Yes
82	mice fed HFD	IR	Yes
83	mice fed HFD	IR	Yes
84	mice fed HFD	IR	Yes
85	mice fed HFD	IR	Yes
86	mice fed HFD	IR	Yes
87	mice fed HFD	IR	Yes
89	mice fed HFD	IR	Yes
90	mice fed HFD	IR	Yes
91	mice fed HFD	IR	Yes
59	mice fed HFD	Not mentioned	Yes, the changes in inflammation phenotypes are due to the changes in obesity
71	Lep^ob/ob^ mice	Not mentioned	Yes, the changes in inflammation phenotypes are due to the changes in obesity
13	obese patients	type 2 diabetes	-
14	obese patients	type 2 diabetes	-
33	obese patients	type 2 diabetes	-
30	obese patients	Not mentioned	-
52	obese Labrador Retriever dogs	IR	-
22	RBP4-Ox mice fed on chow diet	IR	-
35	microenvironment under high levels of insulin in vitro	IR	-
80	iNKT cell-deficient mice	Not mentioned	No, but this article focused on the changes in inflammation phenotypes in adipose tissue
88	Mice lacking γδ T cells or IL-17A	Not mentioned	No, but this article focused on the changes in inflammation phenotype in adipose tissue
92	m Il7r^Cre^Tnfsf4^fl/fl^ mice	Not mentioned	No, but this article focused on the changes in inflammation phenotype in adipose tissue
93	ILC2-deficient mice	Not mentioned	No, but this article focused on the changes in inflammation phenotype in adipose tissue
21	aged mice	IR	No, the IR and inflammation phenotypes are associated with age

IR, insulin resistance; HFD, high fat diet.

## 1. Correlation between treg cells and obesity with IR

In 2009, Feuerer et al [11]. first discovered that the overall transcript profile of the Treg cell population from visceral fat differed from the patterns of spleen and lymph node, and the gene expression differences were not a simple reflection of Treg cell activation. The numbers of Treg cells were strikingly and specifically reduced in insulin-resistant models of obesity. Treg depletion and selectively elicitation revealed that these Treg cells affected the synthesis of inflammatory mediators and glucose uptake by cultured adipocytes by synthesizing anti-inflammatory factors interleukin (IL)-10, thus improving the inflammatory state of adipose tissue and IR. These results unprecedentedly suggest that Treg cells are beneficial to regulate metabolic homoeostasis in adipose tissue. Subsequently, Eller et al [12]. found that compared with those in the normal control group, natural Treg cells were significantly reduced in the visceral adipose tissue of obese patients with IR, suggesting that Treg cells are related to IR. In animal experiments, the use of anti-CD25 monoclonal antibodies to deplete Treg cells enhanced the IR of db/db (leptin receptor-deficient) mice, manifested by increased fasting blood glucose levels, decreased insulin sensitivity, and increased features of diabetic nephropathy (proteinuria and glomerular ultrafiltration). This study confirmed the relationship between Treg cells and IR and suggested the potential role of Treg cells in reversing obesity-related IR and diabetic nephropathy.

In addition to these results in animal experiments, Wu et al [13]. found that the proportion of Treg cells in the omental adipose tissue of obese patients with type 2 diabetes was decreased and that body mass index was negatively correlated with the proportion of Treg cells, suggesting that a decline in the number of Treg cells may be closely related to the occurrence and development of obesity and diabetes. Similarly, Yuan et al [14]. found that the proportion of Treg cells was significantly reduced in a group of obese patients with type 2 diabetes compared with the normal control group, accompanied by apparently decreases in the IL-10 and transforming growth factor-β (TGF-β). Moreover, the proportion of Treg cells was negatively correlated with the degree of IR and positively correlated with the proportion of TGF-β. Because of their anti-inflammatory effects, Treg cells were further confirmed to be an important regulator of obesity-induced IR.

A study by Ilan et al [15]. further emphasized the important protective role of Treg cells in obesity with IR. They found that the increased production of Treg cells in the adipose tissue of ob/ob (leptin-deficient) mice induced by anti-CD3 monoclonal antibodies and β-glucosylceramide reduced liver fat accumulation and significantly decreased blood glucose and liver enzyme levels. More importantly, decreased inflammation was observed in adipose tissue, manifested by the reduced infiltration of CD11b^+^ F4/80^+^ macrophages and a marked decrease in tumour necrosis factor-α (TNF-α) levels. The above results confirmed the close correlation between adipose tissue inflammation and obesity with type 2 diabetes and suggested a unique immunological method to alleviate inflammation and metabolic disorders by inducing increased production of Treg cells in adipose tissue, providing a new direction for the treatment of obesity with IR.

In 2012, Cipolletta et al [16]. identified that peroxisome proliferator-activated receptor (PPAR-γ), the master regulator of adipocyte differentiation, as a crucial molecular orchestrator of adipose Tregs accumulation, phenotype, and function. Moreover, PPAR-γexpression by adipose Tregs was necessary for the complete restoration of insulin sensitivity in obese mice by the thiazolidinedione drug pioglitazone. This research unprecedentedly described the molecular mechanism for the uniqueness of adipose Tregs. Further, Han et al [17]. found that the adipose Tregs of unique phenotype expressed the ST2 chain of the IL-33 R. The proportion of ST2+ Tregs in adipose tissue was severely diminished in obese mice with a high-fat-fed diet compared with lean mice. Moreover, IL-33 treatment completely reversed this effect and decreased the inflammation and IR in adipose tissue of obese mice. This study further explored the important mechanism of adipose Tregs regulating immune homoeostasis, providing a novel direction for the treatment of obesity with IR. However, Bapat et al [18]. found mice deficient in fat-resident Tregs are protected against age-associated IR, yet remain susceptible to obesity-associated IR and metabolic disease. On the contrary, selective depletion of Tregs in adipose tissue via anti-ST2 antibody treatment increased IR. These findings revealed the distinct roles of fat-resident Tregs in ageing- and obesity-associated IR, re-emphasizing Tregs as potential therapeutic targets in the treatment of IR.

## 2. Treg cells regulate the occurrence and development of obesity with IR by interacting with other immune cells

### 2.1 CD4^+^ T effector cells

Naive CD4^+^ T cells settle in secondary lymphoid organs (such as the spleen and lymphocytes) and nonlymphoid organs (such as adipose tissue) after developing in the thymus. By activating antigen-presenting cells (APCs), naive CD4^+^ T cells can further differentiate into T helper cells and T effector cells, the latter of which includes Th1 cells, Th2 cells, Th17 cells, etc. [19,20]. Schmidleithner et al [21]. found that Treg cells can inhibit CD4^+^ T effector cells by specifically upregulating hydroxy prostaglandin dehydrogenase (HPGD), thereby preventing local inflammation and systemic IR. This study suggested that the balance of Treg cells and CD4^+^ T effector cell subsets may play an important regulatory role in obesity with IR.

#### 2.1.1 Th1 cells

In 2014, Moraes-Vieira et al [22]. found that mice overexpressing retinol-binding protein 4 (RBP4) (RBP4-Ox) developed IR and glucose tolerance and exhibited significantly increased macrophage and CD4^+^ T cell infiltration in adipose tissue. Compared with those in the adipose tissue of control mice, macrophages in the adipose tissue of RBP4-Ox mice promoted the proliferation of Th1 cells and the differentiation of naive CD4^+^ T cells into Th1 cells. The expression of interferon-γ (IFN-γ), a characteristic cytokine of Th1 cells, and a CD4^+^ T cell lineage transcription regulator (Tbx21) was also significantly higher than that of the control group, suggesting that RBP4 may cause IR by promoting the proliferation and differentiation of Th1 cells and further aggravating the inflammation of adipose tissue. Rocha et al [23]. further found that the weight of visceral adipose tissue and the expression of pro-inflammatory monocyte chemoattractant protein-1 (MCP-1) in adipose tissue were significantly decreased and that glucose tolerance was significantly improved in obese mice deficient in IFN-γ compared with wild-type mice. Additionally, Zhang et al [24]. found that the anti-IFN-γ antibody could reduce the expression of MCP-1 and IR in obese mice. The above results confirmed that IFN-γ, a cytokine characteristic of Th1 cells, is associated with the occurrence of inflammation and metabolic disorders in adipose tissue, further emphasizing the pro-inflammatory effect of Th1 cells in obesity with IR.

Liu et al [25]. found that the weight of epididymal adipose tissue, fasting blood glucose levels, and insulin levels after high-fat feeding were significantly reduced in T cell costimulatory molecule (OX40)-knockout mice compared with wild-type mice. Moreover, the ratio of Th1/Treg cells was decreased, which indicated that an imbalance of the Th1/Treg cell ratio may be related to the occurrence of OX40-mediated obesity and IR. Furthermore, they found that the expression of Tbx21 and IFN-γ in CD4^+^ T cells in the spleens of OX40-knockout mice was also significantly downregulated, suggesting that the absence of OX40 inhibited the differentiation of CD4^+^ T cells into Th1 cells. Besides, when spleen Treg cells were cocultured with CD4^+^CD25^−^ T cells (T effector cells, Teff cells) at a ratio of 1:4, the Treg cells of OX40-knockout mice had a significantly stronger suppressive effect on Teff cells than those of wild-type mice, suggesting that the absence of OX40 enhanced the suppressive function of Treg cells. The above results confirmed that OX40 deficiency can prevent the occurrence of obesity with IR by rebalancing the Th1/Treg cell ratio, further emphasizing the important role of balance of the Th1/Treg cell ratio in maintaining the metabolic homoeostasis of adipose tissue.

Similarly, Priceman et al [26]. found that functional knockout of the signal transducer and activator of transcription 3 (Stat3) gene in T cells significantly improved insulin sensitivity and glucose tolerance and inhibited the expression of related inflammatory factors, suggesting that Stat3 in T cells mediates adipose tissue inflammation, obesity, and IR. Additionally, compared with Stat3^+/+^ mice, Stat3^−/-^ mice expressed fewer IFN-γ in adipose tissue, and the proportion of Treg cells was significantly increased, suggesting that Stat3 deficiency reduced the Th1/Treg cell ratio. In contrast, in vitro experiments further found that the proliferation and differentiation of Treg cells in the adipose tissue of Stat3^+/+^ mice were significantly decreased after high-fat feeding, suggesting that the expression of stat3 can inhibit the proliferation of Treg cells. The above results revealed that the imbalance of the Th1/Treg cell ratio in adipose tissue is an important mechanism of metabolic disorder of adipose tissue mediated by Stat3 in T cells, again emphasizing that balance of the Th1/Treg cell ratio is conducive to the prevention of inflammation and IR in obese mice.

#### 2.1.2 Th17 cells

As Th17 cells are generally considered pro-inflammatory cells [27–29], the inflammatory state of obese patients with IR may be associated with the increased infiltration of Th17 cells in adipose tissue. Vega-Cárdenas et al [30]. found that compared with those in the adipose tissue of the normal group, Th17 cells in the adipose tissue of obese patients were significantly increased, and the supernatant of cultured monocytes also contained more IL-17, a cytokine characteristic of Th17 cells. Furthermore, Eljaafari et al [31]. found that the adipose-derived stem cells of obese patients were more likely to promote the differentiation of naive T cells into Th17 cells. Together, the above results suggest that Th17 cells are related to obesity-induced inflammation of adipose tissue. In addition, RORγ is selectively expressed on Th17 cells and can be used to identify the phenotype of Th17 cells [32]. Clinical data showed that the mRNA expression of RORγ in the adipose tissue stromal vessels was positively correlated with the size of adipocytes and IR levels in obese patients [33]. Nevertheless, Meissburger et al [34]. found that the body weight, size of adipocytes, and blood glucose and IR levels after 8 weeks of a high-fat diet were significantly decreased in RORγ^−/-^ mice compared with wild-type mice, further emphasizing that Th17 cells mediate the development of obesity with IR.

More importantly, Martinez-Sanchez et al [35]. found that stimulation with high levels of insulin significantly increased the number of naive CD4^+^ T cells that differentiated into Th17 cells while dramatically decreasing their differentiation into Treg cells in vitro. Imbalance of the Th17/Treg cell ratio has been suggested to serve as an important mechanism of obesity with hyperinsulinemia. Furthermore, Gilleron et al [36]. found that the absence of Ras-associated GTP-binding protein 4b (Rab4b) in the T cells of adipose tissue could promote the occurrence of inflammation and IR. More importantly, in vitro, only approximately 30% of Th0 cells in mice with T cell-specific Rab4b KO differentiated into Treg cells and 3-fold more Th17 cells were present in these mice compared with mice in the normal group. However, nearly 40% of Th0 cells in control mice differentiated into Treg cells, while merely about 5% of Th0 cells differentiated into Th17 cells. This result confirmed that the imbalance of the Th17/Treg cell ratio is an essential mechanism for the metabolic disorder in adipose tissue mediated by the absence of Rab4b in T cells.

Similarly, Li et al [37]. found that overexpression of protein tyrosine phosphatase N 2 (PTPN2) in epididymal fat tissue significantly improved inflammation and IR in obese mice. Stat3 is a key positive regulator of the characteristic transcription factor RORγt and characteristic cytokine IL-17 in Th17 cells, which can promote the differentiation of Th17 cells [38] and inhibit the differentiation of Treg cells [39,40]. They found that the activated Stat3 (P-Stat3) level was increased by 51% in diabetic mice compared with control mice, whereas the overexpression of PTPN2 reduced the level of P-Stat3 by 38%, accompanied by a significant decrease in Th17 cells and an obvious increase in Treg cells. The overexpression of PTPN2 was suggested to inhibit the differentiation of Th17 cells and promote the differentiation of Treg cells by downregulating Stat3, which further confirmed that an imbalance of the Th17/Treg cell ratio can mediate the occurrence of obesity-related inflammation and metabolic disorders.

### 2.2 CD8^+^ T cells

CD8^+^ T cells play a significant role in chronic infection and cytotoxic T cell immunity in tumours [41–43], but their effects on obesity-related metabolic disorders remain to be further elucidated. Nishimura et al [44]. found that the epididymal adipose tissue of mice fed a high-fat diet had been infiltrated by a large number of CD8^+^ T effector cells. Similarly, Revelo et al [45]. found that compared with wild-type mice, perforin-knockout mice had increased insulin levels and IR, accompanied by a marked increase in the infiltration of CD8^+^ T cells into visceral adipose tissue. These studies suggested that CD8^+^ T cells are closely related to metabolic disorders in adipose tissue. Additionally, Revelo et al [45]. found that the adoptive transfer of CD8^+^ T cells aggravated inflammation and IR in the adipose tissue of obese mice. In contrast, the depletion of CD8^+^ T cells reduced inflammation and IR. This study confirmed that CD8^+^ T cells mediate obesity-related inflammation and IR. Furthermore, both Deiuliis et al [46]. and Hellmann et al [47]. found that CD8^+^ T cells could mediate the occurrence of IR and systemic glucose intolerance by promoting the recruitment and activation of pro-inflammatory macrophages in adipose tissue, which definitively revealed the important mechanism by which CD8^+^ T cells promote the occurrence of metabolic disorders in adipose tissue.

Recently, more studies have reported that the inhibitory effect of Treg cells on CD8^+^ T cells plays an important role in type 1 diabetes [48–50], but its role in IR in adipose tissue has yet to be clarified. In 2011, Deiuliis et al [46]. first discovered that compared with those in the normal mice, the proportion of Treg cells in the adipose tissue of obese mice decreased from 9.5% to 2.3%, while the proportion of CD8^+^ T cells increased by approximately 2-fold. Similarly, Carreras et al [51]. found that the body weight and fasting blood glucose and insulin levels of sleep apnoea model mice were significantly increased, accompanied by a significantly decreased proportion of Treg cells but the increased infiltration of CD8^+^ T cells in visceral adipose tissue. Besides, in 2018, Palatucci et al [52]. confirmed the above phenomena in obese dogs. They found that the number of Treg cells was significantly decreased, while the proportion of CD8^+^ T cells was significantly increased in obese Labrador retrievers compared with Labrador retrievers in the normal weight group. Together, these results showed that a reduction in Treg cells in adipose tissue may promote the pro-inflammatory tendency of visceral adipose tissue in obese mice by reducing the suppression of CD8^+^ T cells.

Furthermore, studies have directly demonstrated the crucial role of the inhibition of CD8^+^ T cells by Treg cells in the metabolic regulation of adipose tissue. Frias et al [53]. found that the transcriptional regulator Id2 is essential for the expression of proteins required for the survival of Treg cells. The Treg cell-specific loss of Id2 in mice resulted in a significant decrease in Treg cells and an increase in CD8^+^ T cells in adipose tissue after a high-fat diet, accompanied by impaired glucose tolerance and increased plasma insulin levels. This study confirmed that the inhibitory effect of Treg cells on CD8^+^ T cells is beneficial to maintain the metabolic homoeostasis of adipose tissue. Eller et al [12]. further found that Treg cell depletion markedly increased the infiltration of CD8^+^ T cells in the kidney and visceral adipose tissue of obese mice and led to the development of IR and diabetic nephropathy. However, adoptive transfer of Treg cells significantly reduced the infiltration of CD8^+^ T cells in the kidney and visceral adipose tissue of obese mice and significantly improved IR. These results confirmed that Treg cells can reduce obesity-related inflammation and metabolic disorders by inhibiting CD8^+^ T cells.

### 2.3 Adaptive immune cells

#### 2.3.1 Macrophages

Macrophages are important immune cells that play a critical role in tissue homoeostasis, inflammation, and pathology [54]. Macrophages are generally characterized as CD11b^+^F40/80^(+)^ cells. As CD11c and Ly6c are inflammatory markers, and CD206 is an anti-inflammatory marker, macrophages can be generally divided into F4/80^(+)^CD206^(-)^ pro-inflammatory M1 macrophages and F4/80^(+)^CD206^(+)^ anti-inflammatory M2 macrophages [55].

Macrophages are the most common immune cells that infiltrate and accumulate in adipose tissue, accounting for up to 40% of the total accumulated immune cells in adipose tissue. In recent years, a large number of studies have found that macrophages in adipose tissue play an important role in the occurrence and development of obesity and IR [56,57]. In 2003, xu et al [58]. found that the inflammation and macrophage-specific genes are dramatically upregulated and the infiltration of macrophages was significantly increased in white adipose tissue in mouse models of genetic and high-fat diet-induced obesity. Further, Weisberg et al [59]. found that the percentage of cells expressing the macrophage marker CD68 was significantly and positively correlated with both adipocyte size and body mass in adipose tissue of obese individuals. Moreover, the adipose tissue macrophages are responsible for almost all adipose tissue TNF-α expression and significant amounts of iNOS and IL-6 expression. The above two studies firstly demonstrated that macrophages in adipose tissue play an active role in morbid obesity and macrophage-related inflammatory activities may contribute to the pathogenesis of obesity-induced IR in both mice and humans.

Moreover, Ying et al [60]. found that macrophages in the adipose tissue of obese mice could secrete exosomes containing microRNAs (miRNAs). Normal mice developed glucose intolerance and IR when injected with these exosomes, suggesting that the miRNAs of macrophages in adipose tissue are closely related to the occurrence of metabolic disorders. Furthermore, Dalmas et al [61]. found that the specific knockout of interferon regulatory factor 5 (IRF5) in macrophages significantly improved glucose tolerance, insulin sensitivity, and insulinemia in mice fed a high-fat diet. Additionally, Ying et al [62]. found that CD11c^+^ pro-inflammatory macrophages in the islets of high-fat diet-fed mice could inhibit the function of islet β cells in vitro, resulting in a marked reduction in insulin secretion by β cells under glucose stimulation. The above two studies have further revealed the important mechanisms of macrophage-mediated obesity with IR.

Treg cells can directly interact with macrophages in tissues and inhibit the activation of T effector cells by blocking the interaction of APCs with CD4^+^ T effector cells [63,64]. Thus, Treg cells may be able to prevent metabolic disorders by interacting with macrophages to reduce the inflammatory response of adipose tissue. Jin et al [65]. found that 1,25-dihydroxy vitamin D3 significantly reduced blood glucose and insulin levels in high-fat diet-fed mice, accompanied by the markedly increased infiltration of Treg cells and an obvious decrease in pro-inflammatory macrophages in pancreatic islets and adipose tissue. The results of this study suggested that due to their anti-inflammatory effect, Treg cells may reduce the infiltration of macrophages in pancreatic islets and adipose tissue. Furthermore, Zhong et al [66]. found that the knockout of costimulatory molecules B7-1 and −2 (CD80 and CD86, respectively) resulted in a significant decrease in the number and proliferation of Treg cells in the adipose tissue of high-fat diet-fed mice, with the excessive infiltration of macrophages in adipose tissue and significantly increased fasting blood glucose and insulin levels. More importantly, they found that the adoptive transfer of Treg cells significantly reduced the infiltration and classical activation of macrophages in adipose tissue and significantly decreased the inflammatory response and IR in B7-knockout mice. In vitro experiments further revealed that Treg cells could significantly inhibit the proliferation and activation of CD11c^+^CD11b^+^ macrophages. The above research results further confirmed that Treg cells play a protective role against obesity-induced IR by inhibiting the proliferation and activation of pro-inflammatory macrophages.

Conversely, Deiuliis et al [46]. found that CD11b^+^CD11c^+^ pro-inflammatory macrophages and CD11b^+^Ly6C^+^ pro-inflammatory macrophages were significantly increased in obese mice compared with normal control mice, while the number of Treg cells decreased from 9.5% in the normal control mice to 2.3% in the obese mice. Moreover, the proportion of Treg cells in visceral adipose tissue was negatively correlated with the number of CD11b^+^CD11c^+^ macrophages, suggesting that macrophages that infiltrate adipose tissue may inhibit the production of Treg cells, thereby promoting the occurrence of obesity. Furthermore, stimulation with IFN-γ and lipopolysaccharide (LPS) caused bone marrow-derived macrophages to exhibit a pro-inflammatory tendency, causing approximately 5% of naive T cells to differentiate into Treg cells. However, nearly 12% of naive T cells differentiated into Treg cells when cocultured with untreated macrophages, which showed that the infiltration of pro-inflammatory macrophages can inhibit the differentiation of Treg cells in local adipose tissue. In summary, Treg cells and pro-inflammatory macrophages antagonize each other, and an imbalance of the number and function of these two cell types may be an important mechanism of obesity with IR.

#### 2.3.2 Dendritic cells

Dendritic cells (DCs) have the unique ability to initiate T cell responses and play a key role in coordinating adaptive immune responses [67]. In both mice and humans, DCs are divided into conventional dendritic cells (cDCs) and plasma cell-like dendritic cells (pDCs) according to differences in their transcription and functions, with the former including CD103 (cDC1) and CD11b (cDC2) subsets [68,69].

In 2012, Bertola et al [70]. were the first to discover that most of the IR-related DCs in the adipose tissue of obese mice are part of a pro-inflammatory CD11C^high^F4/80^low^ DC subpopulation. In contrast, the anti-inflammatory cDC1 subgroup in the adipose tissue of normal mice could promote the proliferation of Treg cells in adipose tissue, providing an anti-inflammatory signal to prevent associated inflammation. The above research results indicated that different DC subsets play disparate roles in the regulation of adipose tissue metabolism, but the cDC1 subgroup can alleviate inflammation in adipose tissue by promoting the proliferation of Treg cells.

Furthermore, Moraes-Vieira et al [71]. found that the number and maturity of DCs in Lep^ob/ob^ (leptin-deficient) mice were significantly reduced compared with those in wild-type mice, accompanied by decreased expression of the inflammatory factors IL-12, TNF-α, and IL-6. In vitro, coculture experiments showed that the DCs of Lep^ob/ob^ mice promoted the proliferation and differentiation of Treg cells. The above results suggested that DCs participate in the regulation of inflammatory responses in obese leptin-deficient mice by mediating the proliferation and differentiation of Treg cells. More importantly, Macdougall et al [72]. found that cDC1 and cDC2 in visceral adipose tissue could activate the Wnt/β-catenin and Pparg pathways, respectively, thereby maintaining the immune stability of adipose tissue. Compared with wild-type mice, both Ctnnb1^−/-^ (specific knockdown of β-catenin in cDCs) mice and Pparγ^−/-^ (specific knockout of Pparγ in cDCs) mice showed significant reductions in IL-10 expression and Treg cells in visceral adipose tissue after 12 weeks of a high-fat diet, with reduced glucose tolerance and increased IR. The above results confirmed that the dysregulation of related pathways in DCs can reduce the infiltration of Treg cells in adipose tissue and thus aggravate the inflammation and IR caused by obesity. Subsequently, they further confirmed that the activation of β-catenin in DCs increased the number of Treg cells in visceral adipose tissue, improved insulin sensitivity, and prevented the occurrence of type 2 diabetes in obese mice [73]. The above research confirmed that the effect of DCs in promoting Treg cells helps maintain the metabolic homoeostasis of adipose tissue and further clarified the key molecular mechanism by which DCs promote the anti-inflammatory phenotype of visceral adipose tissue.

### 2.4 iNKT cells

Natural killer T (NKT) cells possess the characteristics of adaptive T lymphocytes and natural killer (NK) cells and exert immunomodulatory functions through the rapid production of cytokines [74]. CD1d-restricted NKT cells, called iNKT cells or type 1 NKT cells, express a fixed TCR-α chain and are highly expressed in human and mouse adipose tissues [75].

In 2012, Lynch et al [76]. found that iNKT cell-deficient mice developed IR after high-fat diet feeding, and their body weight, adipocyte size, and IR levels were significantly increased. However, adoptive transfer or activation of iNKT cells in obese mice significantly improved insulin sensitivity by producing anti-inflammatory cytokines, such as IL-10. Moreover, Lynch et al [77] further found an iNKT cell-FGF21 axis, a new immune-mediated pathway, which could be targeted for glycaemic control and weight regulation. These results confirmed that iNKT cells in adipose tissue have a protective effect against diet-induced obesity and IR. In addition to a protective role in obesity with metabolic disorders, adipose tissue-resident iNKT cells could direct interplay with adipocytes and prevent insulin resistance under low-fat diet conditions [78]. LaMarche et al [79]. further revealed that NK1.1^NEG^ iNKT cells dominantly produce IL- 10, driven by intracellular lipid accumulation and IRE1a-XBP1s signalling. Conversely, NK1.1^POS^ cells produce IFN-γ, which, in lean adipose tissue, drives NK cell-mediated macrophage killing to limit pathogenic macrophage expansion. These studies confirm that iNKT cells contribute to the metabolic homoeostasis of adipose tissue in both healthy and obese states. More importantly, Lynch et al [80]. treated high-fat diet-fed mice with sphingolipids (αGalCer), which effectively activated iNKT cells when presented by CD1d on APCs [81]. They found that three days after treatment with αGalCer, the number of Treg cells in the adipose tissue of wild-type mice was significantly increased, suggesting that the activation of iNKT cells promoted the proliferation and activation of Treg cells. In contrast, the proportion and proliferation of Treg cells in adipose tissue were significantly reduced in both CD1d^−/-^ and Ja18^−/-^ (iNKT cell-deficient) mice compared with wild-type mice. The above results confirmed that iNKT cells can prevent the occurrence of obesity-related IR by promoting the proliferation of Treg cells in adipose tissue.

However, the role of iNKT cells in obesity-related inflammation and IR remains controversial. Wu et al [82]. found that iNKT cell deficiency improved adipose tissue inflammation and prevented obesity-induced IR and liver steatosis. In contrast, iNKT cell agonists were found to exacerbate obesity-related inflammation and metabolic disorders. The above findings are contrary to the conclusions of Lynch et al [76]. More importantly, Wu et al [82]. found that after the treatment of obese mice with αGalCer, the proportion of Treg cells in their adipose tissue was significantly reduced, and tissue inflammation, IR, and hepatic steatosis were aggravated. The above results suggested that iNKT cells can mediate the occurrence of obesity-related inflammation and metabolic disorders by inhibiting the production of Treg cells. Although these conclusions conflict, the above studies have confirmed that iNKT cells are involved in regulating metabolic homoeostasis in adipose tissue by acting on Treg cells.

### 2.5 Eosinophils

In 2011, Wu et al [83]. found that the body fat and fasting glucose were increased in ΔdblGATA (eosinophil-deficient) mice with high-fat feeding, and IR and glucose tolerance was significantly impaired. Conversely, the eosinophils of ΔdblGATA mice increased significantly after infection with parasites, with decreased fasting glucose and improved glucose metabolism homoeostasis. Lee et al [84]. further found that eosinophils promote the differentiation and maturation of adipocytes. The above results together suggested that eosinophils are beneficial to maintain the metabolic homoeostasis of adipose tissue. Also, Bolus et al [85]. found that compared with wild-type mice, glucose tolerance in adipose tissue of CCR2-/- mice was improved, and the number of Foxp3 and eosinophils in epididymal adipose tissue was increased, and the numbers of the two were positively correlated, suggesting that eosinophils might increase the expression of Treg cells in adipose tissue. However, more studies are needed to confirm that the interaction between the above two cells regulates the metabolic homoeostasis of adipose tissue.

### 2.6 γδ T cells

In 2015, Tougaard et al [86]. found that γδT+ cells were significantly reduced in adipose tissue of mice with improved glucose metabolism. Mehta et al [87]. further found that compared with wild-type mice, TCRδ-/- (γδT cell-deficient) mice had significantly lower pro-inflammatory macrophages, CCL2, and IL-6 in epididymal adipose tissue, while the clearance rate of blood glucose increased significantly. The above results confirmed that γδ T cells promote inflammation and glucose metabolism disorders in adipose tissue. At present, a few research have shown that γδT cells can regulate obesity-related IR through interaction with Treg cells. However, Kohlgruber et al [88]. found that at 20 weeks of age, the proportion of Treg cells in adipose tissue of TCRδ-/- mice was significantly lower than that of wild-type mice, and the expression of IL-10 and KLRG1 were also significantly decreased, suggesting that γδT cells can promote the accumulation of Treg cells in visceral adipose tissue in age-related IR. However, the accumulation of fat-resident Tregs exacerbated age-associated IR [18]. Thus, depletion of γδT cells may contribute to both obesity and age-associated IR.

### 2.7 ILC2s

In 2013, Molofsky et al [89]. found that the deficient of ILC2s caused a significantly decreased IR in high-fat-fed mice. Hams et al [90]. found that the adoptive transfer of ILC2s reduced the body weight and stabilized the glucose metabolism of obese mice. The above studies confirmed that ILC2s are beneficial to maintain the immune homoeostasis of adipose tissue. Also, Brestoff et al [91]. found that IL-33 increased the number of ILC2s in adipose tissue, reduced the body weight and fat mass, and improve glucose homoeostasis of high-fat-fed mice, suggesting that the IL-33/ILC2 pathway may be a novel way to treat obesity and metabolic disorders. More importantly, Halim et al [92]. found that IL-33 promoted the expansion of Treg cells, and the expansion of Treg cells was significantly reduced after the targeted knockout of OX40L in ILC2s. Similarly, Molofsky et al [93]. found that in ILC2-deficient mice, the expansion of Treg cells induced by IL-33 was significantly impaired. The above results all confirmed that the homoeostasis regulation of Treg cells by IL-33 depends on ILC2s. Also, Halim et al [92]. further found that ILC2 promoted the proliferation and activation of KLRG1+ IL1RL1+ Treg cells in adipose tissue through the interaction between ICOSL-ICOS, further confirmed the critical regulatory role of ILC2s to Treg cells in adipose tissue, and revealed the important mechanism of IL-33/ILC2s regulating the metabolic homoeostasis of adipose tissue.

## 3. Conclusion and outlook

The prevalence of obesity-induced IR and type 2 diabetes has remained high in recent years and is rapidly increasing [94], and Treg cells play an important protective role in obesity with IR. Treg cells reduce inflammation in adipose tissue by secreting the anti-inflammatory cytokine IL-10, TGF-β, whose expression of PPAR-γ is also essential for maintaining IR in adipose tissue. Recent studies suggest that IL-33/ST2 gets a new direction in the treatment of obesity with IR. More importantly, the interactions between Treg cells and other immune cells play an important role in obesity-induced or related IR. Treg cells are antagonistic to Th1 and Th17 cells respectively, and the imbalanced ratio of Treg cells to Th1 or Th17 cells will lead to the occurrence of metabolic disorders in adipose tissue. Stat3 can both promote the secretion of Th1 and Th17 cytokines, so Stat3 may be a protein target for IR treatment. Pro-inflammatory macrophages can also antagonize Treg cells, and the imbalance of their functions and numbers is one of the essential cellular mechanisms of obesity with IR. In addition, Treg cell-specific Id2 deficiency alleviates IR in adipose tissue by inhibiting CD8 + T cells. However, anti-inflammatory dendritic cells, eosinophils, and ILC2s can help prevent obesity-associated IR by promoting the differentiation of Treg cells, especially the IL33/ILC2s pathway, which is beneficial for the metabolism regulation of adipose tissue. The effect of iNKT cells on Treg cells is contradictory and consistent with their roles in obesity with IR. iNKT cells can promote the occurrence of metabolic disorders by inhibiting the differentiation of Treg cells. Conversely, they can also maintain the metabolic homoeostasis of adipose tissue by promoting the differentiation of Treg cells. Further studies are needed to clarify the role of iNKT cells in obesity with IR. γδT cells mediate the occurrence of obesity combined with IR, and can also promote age-related IR by increasing the infiltration of Treg cells in adipose tissue, emphasizing the different roles of Treg cells in obesity and age-related IR. Thus, maintaining a balance of the numbers and functions of Treg cells and other immune cells will be a new way to treat obesity and IR. However, the molecular mechanisms by which the interactions between Treg cells and various immune cells are regulated are not fully understood, and more in-depth research is needed to provide a more accurate direction for the treatment of obesity and obesity-related metabolic diseases.

## Data Availability

The authors confirm that the data supporting the findings of this study are available within the article [and/or] its supplementary materials.

## Abbreviations

IR, insulin resistance; Treg, Regulatory T; iNKT, Invariant natural killer T; APCs, antigen-presenting cells; FOXP3, transcription factor forkhead box protein P3; IL, interleukin; TGF-β, transforming growth factor-β; TNF-α, tumour necrosis factor-α; HPGD, hydroxy prostaglandin dehydrogenase; RBP4, retinol-binding protein 4; IFN-γ, interferon-γ; MCP-1, monocyte chemoattractant protein-1; Stat3, signal transducer and activator of transcription 3; PTPN2, protein tyrosine phosphatase N 2; LPS, lipopolysaccharide; DCs, Dendritic cells; ILC2s, type 2 innate lymphoid cells; PPAR-γ, peroxisome proliferator-activated receptor-γ.
